# Soil microbiome analysis of cultivated tomato ( *Solanum lycopersicum* ) plants

**DOI:** 10.17912/micropub.biology.001225

**Published:** 2025-05-23

**Authors:** Nicolas L. Cerioni, Harrison L. Uhl, Mara A. Welty, Jacob J. Adler

**Affiliations:** 1 Department of Biological Sciences, Purdue University, West Lafayette, Indiana, United States

## Abstract

Microbial biodiversity is critical to tomato plant health. The symbiotic relationship between tomato plants and their soil microbiome influences the plants’ ability to absorb nutrients and adapt to environmental stresses. This study compared the soil microbiome between tomato plants appearing healthy versus those appearing unhealthy. There were no significant differences in overall bacterial biodiversity between the conditions. However, a specific beneficial genus ( *Sphingomonas* ) and its phylum Proteobacteria (Pseudomonadota) were found at significantly higher amounts in healthy plants’ soil compared to unhealthy plants’ soil. Our findings show the need for further examination of the benefits of *Sphingomonas* for tomato plants.

**Figure 1. Bacterial Biodiversity in Healthy and Unhealthy Tomato Plants:**
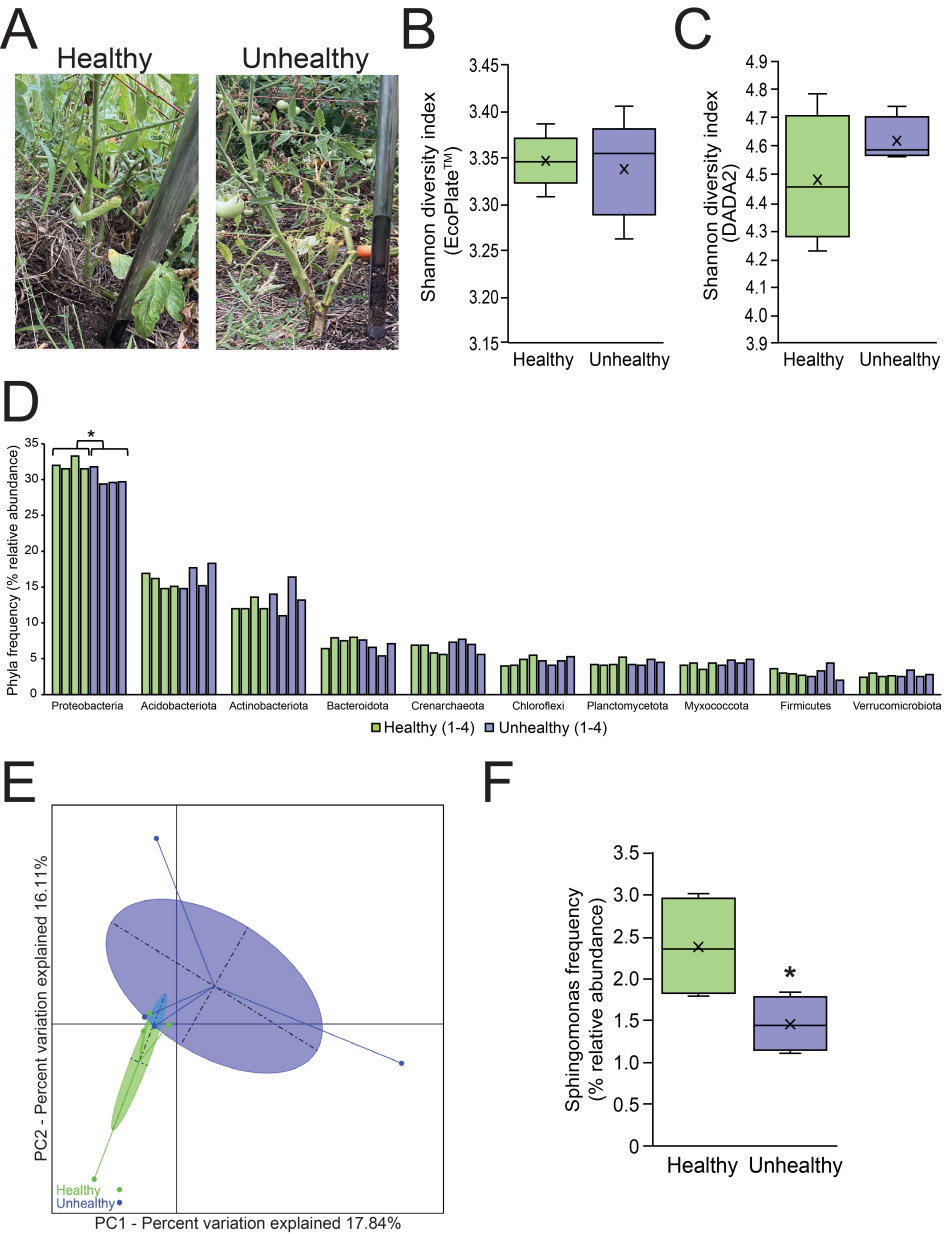
A) Tomato plant soil sites. Representative images of a healthy tomato soil sample site (left) and of an unhealthy tomato soil sample site (right). Healthy tomato plants were characterized by green leaves and stems that were upright and showing signs of growth. Unhealthy tomato plant sites were characterized by wilting leaves and stems with a portion of them, varying in color from yellow/tan to brown. B) Functional biodiversity via EcoPlate ^TM^ analysis. EcoPlate ^TM^ data derived from unique soil samples from healthy (N = 7) and unhealthy tomato plant soil (N = 7) were analyzed. The Shannon diversity index were calculated from this data and reported as box plots. An unpaired assuming unequal variance ttest was performed to compare the two conditions. p-value > 0.05. C) Bacterial alpha diversity via 16S rRNA sequencing analysis. Genomic DNA (gDNA) was extracted from unique soil samples and sequenced with a MiniSeq approach. FASTQ sequence files were then analyzed in Nephele via the DADA2 pipeline. Data derived from unique soil samples from healthy (N = 4) and unhealthy tomato plant soil (N = 4) were analyzed meeting the quality control for gDNA. Shannon diversity index was calculated from this data and reported as box plots. An unpaired assuming unequal variance ttest was performed to compare the two conditions. p-value > 0.05. D) Bacterial phyla biodiversity via 16S rRNA sequencing analysis. Genomic DNA (gDNA) was extracted from unique soil samples and sequenced with a MiniSeq approach. FASTQ sequence files were then analyzed in Nephele via the QIIME2.0 pipeline. The top 10 phyla by percent relative abundance are presented here of those found in the 4 unique sample sites of healthy tomato plant and the 4 unique sample sites of unhealthy tomato plant soil. An unpaired assuming unequal variance ttest was performed to compare the two conditions for each phylum. * = p-value < 0.05. All other phyla comparisons have a p-value > 0.05. E) Bacterial beta diversity via 16S rRNA sequencing analysis. Genomic DNA (gDNA) was extracted from unique soil samples and sequenced with a MiniSeq approach. FASTQ sequence files were then analyzed in Nephele via the DADA2 pipeline. Genomic data derived from unique soil samples from healthy (N = 4) and unhealthy tomato plant soil (N = 4) were analyzed. Principle coordinate analysis was performed. Statistical tests were conducted in R. Adonis test was used to determine the p-value with 999 permutations. p-value > 0.05. F) Bacterial genus *Sphingomonas* percent relative abundance via 16S rRNA sequencing. Genomic DNA (gDNA) was extracted from unique soil samples and sequenced with a MiniSeq approach. FASTQ sequence files were analyzed in Nephele via the DADA2 pipeline. The box plot compares the percent relative abundance of the genus *Sphingomonas* from healthy tomato plant soil (N = 4) and unhealthy soil (N = 4) sites. An unpaired assuming unequal variance ttest was performed to compare the two conditions. * = p-value < 0.05.

## Description

Soil microbial biodiversity is a principal factor in the health and the growth of plants (Tilak et al., 2005). Tomato plants require a diverse and unique presence of microbial organisms and nutrients to optimize the growth of fruit (Bona et al., 2017). Certain factors such as soil pH, moisture content, physical composition, and microbial presence all play a role in supporting plants in their growth (Usharani KV et al., 2019). Depending upon the bacteria taxa present in the soil, increased microbial biodiversity can be beneficial to the plants' health and growth (H. Liu et al., 2017; Saleem et al., 2019).

Here we examined the soil microbial biodiversity of tomato plants grown in soil at a local urban community garden collected on the same day towards the end of the growing season ( Figure 1A ). Plants that held a true green with little to no wilt and had attributions, such as tomato stems, that showed the production of fruit were our healthy samples. Plants that showed wilted leaves, and had brown/tan leaves, with discolorations were considered our unhealthy samples. Visualization alone is not ideal for categorizing plant phenotype as many different variables could contribute to this phenotype. To combat this possible bias, we only utilized plants in the same area of the garden. Further, we had three independent researchers verify the identification of phenotype prior to soil collection. In this study, we wanted to see if the soil microbiome or soil properties were altered when comparing the two associated tomato plant soil phenotypes.

We first examined the soil's pH, moisture content, and overall composition and texture as these have been shown to influence plant health and soil microbial biodiversity (Garbeva et al., 2004; Griffiths et al., 2011; Lange et al., 2014). The mean average pH (+/- standard deviation) of the healthy tomato plant soil (7.82 +/- 0.50) and unhealthy tomato plant soil (7.87 +/- 0.34) were not significantly different (p-value = 0.81). Although both pH readings were slightly higher than the ideal range for tomato plants (pH = 6.2-6.8) for sufficient fruit growth and plant health (Bangera et al., 2023).

Ground conditions need to maintain a certain moisture content for proper plant growth (Seneviratne et al., 2010). Tomato plants grow most efficiently in a soil moisture of 41-80% (Bangera et al 2023). By locking the moisture into the soil, tomato plants can adequately absorb the water needed to maintain life (J. Liu et al., 2019). The mean average soil moisture content (+/- standard deviation) of the healthy tomato plant soil (23.0 +/- 10.0%) and unhealthy soil condition (19.9 +/- 8.8%) were not significantly different (p-value = 0.52). While these moisture content values were lower than recommended, this can likely be attributed to the study occurring during the end of a growing season under drought-like conditions.

The soil composition is another key factor that helps determine soil biodiversity (Garbeva et al., 2004). Therefore, the soil's physical composition was examined and appeared via eye and texture to consist of the same clay soil for both phenotypes. No differences were observed. Overall, there does not appear to be a difference in the three physical characteristics of soil that were examined in our study.

We then examined the functional biodiversity of the soil sites using an EcoPlate ^TM^ . The EcoPlate ^TM^ detects active metabolism of specific carbon course utilization by heterotrophic bacteria in the soil samples (Stefanowicz, 2006). This carbon source utilization data was used to calculate the Shannon diversity index, richness, and evenness of the two soil conditions as described in (Németh et al., 2021). The mean average (+/- standard deviation) Shannon diversity index value of healthy tomato plant soil (3.35 +/- 0.03) and unhealthy tomato plant soil (3.34 +/- 0.05) were not significantly different (p-value = 0.71) ( Figure 1B ). The mean average richness value of healthy tomato plant soil (29.29 +/- 0.76) and unhealthy tomato plant soil (29.43 +/- 1.51) were also not significantly different (p-value = 0.83). Finally, the mean average evenness value of healthy tomato plant soil (0.99 +/- 0.0033) and unhealthy tomato plant soil (0.99 +/- 0.0053) were also not significantly different (p-value = 0.14). Further, there were no observable overall differences in the utilization of five different carbon source categories (amino acids, amines, carbohydrates, carboxylic acids, and polymers) by the soil microbes between the two soil conditions. Samples were taken during drought-like conditions, which could play a contributing role to the lack of variation in functional biodiversity in the soil samples.

Finally, bacterial biodiversity was examined in the soil samples by extracting genomic DNA (gDNA) from the soil and then using a previously described two-staged PCR amplification protocol (Naqib et al., 2018) to specifically isolate 16S ribosomal RNA (rRNA). These amplicons were then sequenced and FASTQ files were generated. See methodology for details. DADA2 (Callahan et al., 2016) and QIIME 2.0 (Bolyen et al., 2019) downstream analyses were performed on the FASTQ files using the platform Nephele (NIH). The QIIME 2.0 analysis produced taxa bar plots (barplot.qzv) of the taxonomies of the samples.

The alpha diversity measures of Shannon diversity index, richness, and evenness came from the DADA2 pipeline and showed similar trends to those of the EcoPlate ^TM^ results. The average (+/- standard deviation) Shannon diversity index of healthy tomato plant soil (4.48 +/- 0.23) and unhealthy tomato plant soil (4.62 +/- 0.08) were not significantly different (p-value = 0.20) ( Figure 1C ). The average richness of healthy tomato plant soil (135.75 +/- 24.81) and unhealthy tomato plant soil (135.75 +/- 12.50) were also not significantly different (p-value = 0.15). Finally, the mean average evenness value of healthy tomato plant soil (0.91 +/- 0.01) and unhealthy tomato plant soil (0.92 +/- 0.005) were also not significantly different (p-value = 0.53).

The top 10 most abundant phyla based upon the percent relative abundance determined via the QIIME 2.0 pipeline were displayed ( Figure 1D ). The percent relative abundance of most phyla displayed no major differences between the soil conditions for the samples. Interestingly, Proteobacteria (Pseudomonadota) was the only top 10 phylum that showed significantly higher frequency in healthy tomato soil versus unhealthy (p-value = 0.03). This phylum contains 9 unique bacteria taxa shown to play significant roles in tomato plant health and will be described here later.

The rarefaction curves from the DADA2 pipeline indicate that our conditions were sampled evenly. The two conditions were then examined for beta diversity ( Figure 1E ). The adonis test with 999 permutations using relative abundance demonstrated no significant difference between the healthy and unhealthy tomato plant soil conditions (p-value = 0.43). These results are consistent with our Shannon diversity indices ( Figure 1B and 1C) and the phyla taxonomic analysis ( Figure 1D ) and suggest that both types of tomato plants have similar overall microbial community structure in the soil.

The overall taxonomy counts (otu_summary_table.txt) were searched in the DADA2 pipeline for specific bacteria taxa that were of interest because of their reported beneficial or harmful effects on the tomato plant. The following beneficial bacteria were searched based upon previous reports: *Paenibacillus polymyxa* (Zhou et al., 2021) *,* *Paenibacillus* *xylanexedens* (Zhou et al., 2021) *,* *Bacillus* *velezensis* (Zhou et al., 2021) *,* *Bacillus* *endophyticus* (Zhou et al., 2021) *,* *Bacilluscabrialesii* (Zhou et al., 2021), *Bacillus* *subtilis (Raji & Thangavelu, 2021; Zhou et al., 2021)* , *Bacillus* *cereus* (Raji & Thangavelu, 2021), *Bacillus* *licheniformis* (Raji & Thangavelu, 2021), *Burkholderia* *cenocepacia* (Raji & Thangavelu, 2021), and *Sphingomonas* (Khan et al., 2014). The following harmful bacteria were searched based upon previous reports: *Xanthomonas* *campestri* (El-Hendawy et al., 2005), *Pseudomonas* *syringae* (Zhao et al., 2003), *Clavibacter* *michiganensis* (Nandi et al., 2018), and *Ralstonia* *solanacearum* (Brown & Allen, 2004). Interestingly, all the researched beneficial and harmful bacteria belong to three phyla: Firmicutes (Bacillota) (10 taxa), Proteobacteria (Pseudomonadota) (9 taxa), and Actinobacteriota (Actinomycetota) (1 taxa). Most of these bacteria were not present in the DADA2 analyses. One genus that was present in our soil samples was *Sphingomonas.* Interestingly, there was a significantly higher percent relative abundance of the *Sphingomonas* in the healthy tomato plant soil compared with the unhealthy tomato plant soil (p-value < 0.05) ( Figure 1F ). A similar trend was observed in the QIIME 2.0 analysis of the *Sphingomonas* percent relative abundance. The other genera found in the soil samples — *Pseudomonas and Bacillus* — showed no statistical differences in their percent relative abundance between the two soil conditions based on the DADA2 pipeline analysis.

*Sphingomonas* is a bacteria that, when present in the soil of tomato plants, produces gibberellins and indole 3-acetic acid, plant growth regulators that can increase the tomato plant growth (Khan et al., 2014). The effect that the presence of *Sphingomonas* can have on tomato plant growth is significant. The Khan et al. study showed that tomato plants treated with *Sphingomonas* increased their shoot length and dry weight and also the root dry weight (Khan et al., 2014). In our study, *Sphingomonas* counts were found at a higher frequency in the healthy tomato plant soil condition compared with the unhealthy plant soil condition ( Figure 1F ). Interestingly, *Sphingomonas* belongs to the phyla Proteobacteria (Pseudomonadota), the only top 10 phyla by relative abundance to have a significant difference between healthy and unhealthy soil conditions. Previous work with tomato plants have demonstrated Proteobacteria enrichment in treatments designed to promote healthy soil and tomato plant growth (Liao et al., 2021; Sun et al., 2022). Further, increased Proteobacteria in soil can suppress pathogens and diseases like those that cause tomato wilt (Raaijmakers et al., 2009; Toyoda, 1988). This research seems to point to an importance for the Proteobacteria phylum in terms of overall health of tomato plants. It may be interesting to research this phylum further for other genera that may be beneficial or unique for these healthy tomato plants.

Together, this work indicates that the bacterial genus, *Sphingomonas* , and its phylum, Proteobacteria, could be playing a role in the health of tomato plants possibly by increasing the number of plant growth regulators in the plant. Future research should examine if there is a direct connection between the health of tomato plants and *Sphingomonas* in the soil microbiome. Larger amounts of the *Sphingomonas* bacteria in tomato plant soil could lead to more growth and increased yields in tomato plants. This points to a possible direction for future care for tomato growers.

## Methods

**Soil Collection**

The soil was collected at the Erie Street Community Sharing Garden run by the local nonprofit GrowLocal ^TM^ in Lafayette, IN, USA on the same day in September 2023. Two different soil corers were used for the two different soil conditions: healthy and unhealthy tomato plant soil; the soil was collected near the base of the stem with a soil depth of approximately 6 inches. The unhealthy tomato plant condition was characterized as the last row of tomato plants in the garden. It contained withered tomato plants with leaves that were tan in color and less fruit than the healthy tomato plants. The healthy tomato plant condition was characterized by their green color, more plentiful fruit, and greater number of leaves. Samples were taken from seven different unique sites for each soil condition.

**Soil Property Tests**

The pH of the soil conditions was determined using a pH meter. Approximately 3 grams of soil were diluted in 12 milliliters of deionized water and vortexed before the reading were obtained via a pH meter. The soil content moisture was determined by measuring the mass of approximately 1 gram of soil which was set in a laboratory oven at 110 °C for 1 week. The soil moisture content was determined via the formula soil moisture = (dry soil weight – wet soil weight) / (dry soil weight) X 100. The conditions were compared for seven unique samples for each condition. An unpaired assuming unequal variance ttest was used to compare the conditions.

**Functional Biodiversity**

Functional biodiversity was determined using a carbon source utilization assay with EcoPlate ^TM^ (Biolog, 1506) via the manufacturer's directions. The delta absorbance at 595 nm (Day 7-Day 0) was calculated after removing background absorbance. Delta absorbance readings over the 0.25 threshold were considered a positive read. The number of unique carbon source positive reads were used to calculate the richness (S) values. Shannon Index values (H) were calculated via the formula H = −Σ [p _i_ × ln(p _i_ )]. The calculated p _i_ = the ratio of delta absorbance for one carbon source divided by the total delta absorbance for all carbon sources. Evenness (E) was calculated with the formula E = H/lnS. Carbon source utilization efficiency was calculated via sum of the positive reads for each carbon source divided by the total positive reads of carbon sources for that condition. H, S, and E values were compared for the two conditions via an unpaired assuming unequal variance ttest.

**16S rRNA Sequencing Analysis**

Genomic DNA (gDNA) was extracted from soil samples using the ZymoBIOMICS ^TM^ DNA Miniprep Kit (Zymo Research, D4300) according to the manufacturer's directions. Spectrophotometry was used to determine the concentration and the 260/280 and 260/230 nm ratios of the gDNA using a BIOSPEC Nano (Shimadzu, 206-26300-42). Four gDNA samples of both healthy tomato plant soil and unhealthy tomato plant soil that met spectrophotometry standards were PCR amplified with primers sIDTP5_515F and sIDTP7_806R (modified from the primer set employed by the Earth Microbiome Project (EMP; CTACACGACGCTCTTCCGATCT GTGYCAGCMGCCGCGGTAA and CAGACGTGTGCTCTTCCGATCT GGACTACNVGGGTWTCTAAT, respectively – underlined regions represent linker sequences) targeting the V4 region of microbial small subunit ribosomal RNA genes. Amplicons were generated using a two-stage PCR amplification protocol like that described previously (Naqib et al., 2018). The primers contained 5’ common sequence tags (sIDTP5 and sIDTP7) that match 3’ sequences present in IDT xGen™ Amplicon UDI primers (part numbers: 10009846, 10009851, 10009852, and 10009853). First stage PCR amplifications were performed in 10 microliter reactions in 96-well plates, using repliQa HiFi ToughMix (Quantabio). Genomic DNA input was 1 microliter per reaction. PCR conditions were 98 °C for 2 minutes, followed by 28 cycles of 98 °C for 10 seconds, 52 °C for 1 second and 68 °C for 1 second. Subsequently, a second PCR amplification was performed in 10 microliter reactions in 96-well plates using repliQa HiFi ToughMix. Each well received a separate primer pair containing unique dual indices (i.e., from the IDT xGen™ amplicon UDI primer sets). One microliter of PCR product from the first stage amplification was used as template for the 2 ^nd^ stage, without cleanup. Two microliters of primer were used per reaction. Cycling conditions were 98 °C for 2 minutes, followed by 8 cycles of 98 °C for 10 seconds, 60 °C for 1 second and 68 °C for 1 second. Libraries were pooled, purified using a 0.6X AMPure (Beckman-Coulter) cleanup, and sequenced with a 10 % phiX spike-in on an Illumina Miniseq sequencer employing a mid-output flow cell (2x154 paired-end reads). Library preparation, pooling, and sequencing were performed at the Genomics and Microbiome Core Facility (GMCF) at Rush University. These paired-end FASTQ files were then merged to create one FASTQ file. Pre-processing quality control was run on the FASTQ files via Nephele (Weber et al., 2018). Then DADA2 (Callahan et al., 2016) and QIIME 2.0 (Bolyen et al., 2019) downstream analyses were performed. QIIME 2.0 produced taxa barplots (barplot.qzv) of the taxonomies of the samples with counts above the sampling depth. DADA2 produced an otu_summary_table.txt, which provides summaries of the sequence variant counts by sample used to calculate the percent relative abundance based upon total feature counts. Richness (S) and Shannon index (H) were obtained from the DADA2 pipeline. Evenness (E) was calculated with the formula E = H/lnS.

A taxonomic table of the top 10 most abundant phyla was created from the QIIME 2.0 analysis (Bolyen et al., 2019), which was used to derive the percent relative abundance using the generated feature count percentage. The percent relative abundance was compared between the four samples per condition using an unpaired two-sided t-test assuming unequal variance with a p-value threshold set at 0.05.

For beta diversity analysis, the 16S rRNA FASTQ sequence files were analyzed using packages in R version 4.3.3 (R Core Team, 2024). A table of operational taxonomic unit (OTU) data was pulled from the non-rarefied BIOM file produced by the DADA2 analysis (Callahan et al., 2016) using phyloseq using the ‘otu_table’ function (McMurdie & Holmes, 2013). Community beta diversity using relative abundance data was assessed using the ‘adonis’ function from the ‘vegan’ package with a Bray–Curtis dissimilarity index matrix and 999 permutations with an p-value threshold set at 0.05. The ‘betadiper’ function in the ‘vegan’ (Dixon, 2003) package was used to determine group homogeneity. The generalized Unifrac was visualized using principle coordinate analysis ordination (PCoA).

## Reagents

**Table:** 

**Name**	**Supplier**	**Sequence/Catalog Number**
Soil Samples	Not Applicable	Not Applicable
DI Water	Not Applicable	Not Applicable
pH Meter	Orion Star	A211
Drying Oven	Baxter	DX-41
EcoPlate	Biolog	1506
ZymoBIOMICS Kit	Zymo Research	D4300
BIOSPEC Nano	Shimadzu	206-26300-42
PCR Machine	BIO-RAD	T100 Thermal Cycler
repliQa HiFi ToughMix	Quantabio	95200-025
Primer Forward sIDTP5_515F	Earth Microbiome Project	CTACACGACGCTCTTCCGATCT GTGYCAGCMGCCGCGGTAA
Primer Reverse sIDTP7_806R	Earth Microbiome Project	CAGACGTGTGCTCTTCCGATCT GGACTACNVGGGTWTCTAAT
IDT xGen™ Amplicon UDI primers	Integrated DNA Technologies	Part numbers: 10009846, 10009851, 10009852, and 10009853
AMPure	Beckman Coulter	A63880
PhiX	Illumina	FC-110-3001
MiniSeq Sequencer	Illumina	Not Applicable
Nephele	National Institutes of Health	https://nephele.niaid.nih.gov
